# Interaction between Polyketide Synthase and Transporter Suggests Coupled Synthesis and Export of Virulence Lipid in *M. tuberculosis*

**DOI:** 10.1371/journal.ppat.0010002

**Published:** 2005-09-30

**Authors:** Madhulika Jain, Jeffery S Cox

**Affiliations:** Department of Microbiology and Immunology, University of California, San Francisco, California, United States of America; University of California at Berkeley, United States of America

## Abstract

Virulent mycobacteria utilize surface-exposed polyketides to interact with host cells, but the mechanism by which these hydrophobic molecules are transported across the cell envelope to the surface of the bacteria is poorly understood. Phthiocerol dimycocerosate (PDIM), a surface-exposed polyketide lipid necessary for *Mycobacterium tuberculosis* virulence, is the product of several polyketide synthases including PpsE. Transport of PDIM requires MmpL7, a member of the MmpL family of RND permeases. Here we show that a domain of MmpL7 biochemically interacts with PpsE, the first report of an interaction between a biosynthetic enzyme and its cognate transporter. Overexpression of the interaction domain of MmpL7 acts as a dominant negative to PDIM synthesis by poisoning the interaction between synthase and transporter. This suggests that MmpL7 acts in complex with the synthesis machinery to efficiently transport PDIM across the cell membrane. Coordination of synthesis and transport may not only be a feature of MmpL-mediated transport in *M. tuberculosis,* but may also represent a general mechanism of polyketide export in many different microorganisms.

## Synopsis

Pathogenic microbes have developed sophisticated strategies to evade the human immune system and establish infection. *Mycobacterium tuberculosis,* the causative agent of tuberculosis, exports a wide array of lipid virulence factors to the cell surface and into host cells, where they can interact with the host immune system. A strategy to combat *M. tuberculosis* infections may be to interfere with the bacterium's ability to make and secrete these lipids. In the authors' efforts to understand this process, they have found that synthesis and export of a key lipid virulence factor are coupled. They propose that the synthesis and transport proteins form a complex that promotes efficient lipid export. The coordination of lipid synthesis and export, analogous to co-translational translocation of secreted proteins, may be a general mechanism employed by many different microorganisms to actively transport hydrophobic molecules from cells.

## Introduction

*Mycobacterium tuberculosis,* the causative agent of tuberculosis, has an extraordinary ability to resist the bactericidal mechanisms of the human immune system [1]. The integrity of the complex mycobacterial cell wall and its associated lipids has long been thought to be important for virulence [2–6]. This complex lipid metabolism is reflected in the enormous capacity of *M. tuberculosis* for polyketide lipid synthesis: there are at least 24 different polyketide synthases and numerous lipid modification enzymes encoded in its genome [7]. Recent evidence has shown that surface-exposed polyketides, associated with the outer leaflet of the cell wall, are central to the pathogenesis of *M. tuberculosis* [8–11]. Genes involved in the synthesis and transport of these polyketides are required for bacterial growth and virulence in mice. Furthermore, these lipids have been shown to provide a direct physical barrier to host-induced damage [12], as well as to modulate the immune response to infection by altering cytokine profiles in macrophages [13–15].

**Figure 1:**
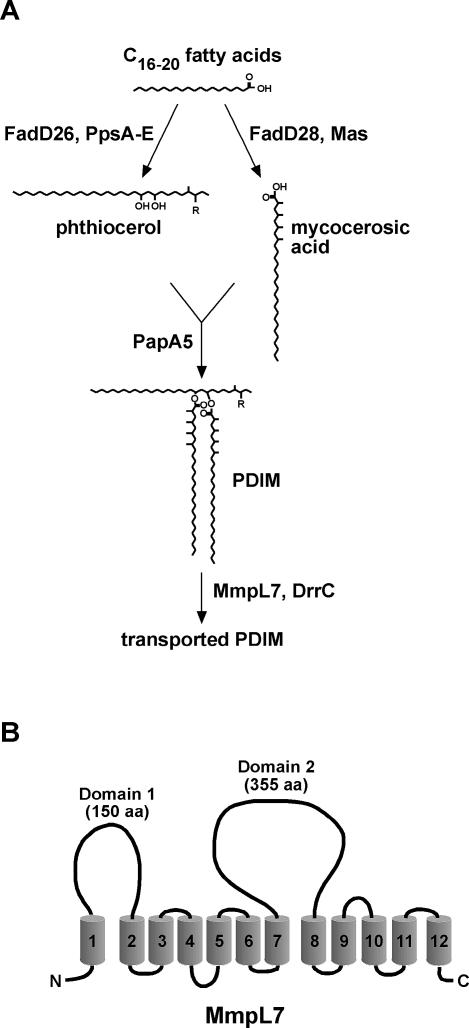
PDIM Synthesis and Export Pathway and Topology of MmpL7 (A) Schematic of the known steps in the PDIM synthesis and transport pathway. PpsA–E and Mas are polyketide synthases that extend fatty acids to phthiocerol and mycocerosic acids, respectively [16,17]. These are then esterified to produce PDIM. MmpL7 and DrrC are required for the transport of PDIM to the cell surface [8,10]. R is =O (keto) or –OCH_3_ (methoxy). (B) Predicted membrane topology of MmpL7 indicating the two non-TM domains 1 and 2.

Many of the surface-exposed polyketides in *M. tuberculosis* require active transport from their site of synthesis in the cytoplasmic membrane to the cell surface. Unfortunately, little is known about the mechanism of polyketide secretion. MmpL7, a member of the MmpL (mycobacterial membrane protein large) family of proteins in *M. tuberculosis,* was the first MmpL shown to be required for the transport of a specific polyketide, phthiocerol dimycocerosate (PDIM) to the cell surface [8]. A number of genes involved in PDIM biogenesis have also been identified [16–18], and the biosynthetic pathway is shown in a schematic in Figure 1A. The PpsA–E gene products extend straight chain fatty acids to phthiocerol, and the mycocerosic acid synthase (Mas) enzyme catalyzes the addition of methyl branches to synthesize mycocerosic acids [16,17,19]. The enzymes FadD26 and FadD28 are thought to be AMP ligases that activate straight chain fatty acids for transfer to the Pps and Mas enzymes [20]. Finally, both MmpL7 and DrrC are required for transport of PDIM to the cell surface [8,10]. MmpL8, another MmpL family member, has been shown to be required for the biogenesis and transport of sulfolipid-1 (SL-1) [9,11]. Given the apparent specificity in MmpL-mediated transport, it is likely that the other eleven MmpL proteins encoded by the *M. tuberculosis* genome transport specific lipids that may be involved in bacterial virulence.

MmpLs belong to a broader class of proteins termed RND (resistance, nodulation, and cell division) permeases [21]. MmpL7, like most RND transporters, is predicted to contain twelve transmembrane (TM) domains with two non-TM domains between TM #1 and #2 (domain 1) and between TM #7 and #8 (domain 2) (Figure 1B). The structure of AcrB, a well studied RND transporter involved in multidrug efflux in *Escherichia coli,* has been determined, and its non-TM domains have been localized to the periplasm [22,23]. AcrB interacts with an outer membrane protein, TolC, to expel a variety of drugs from the cytoplasmic membrane across the entire cell wall [24,25]. Since MmpLs are similar in predicted topology to RND permeases, and are required for the transport of cytoplasmic polyketide substrates, we reasoned that they may also interact with other proteins that are involved in the transport of lipids to the cell surface. To better understand the transport of PDIM to the outer leaflet of the cell wall, we probed for interactors of MmpL7. Surprisingly, we found an interaction between MmpL7 domain 2 and the PDIM synthetic enzyme PpsE that is required for the final step of phthiocerol synthesis. Because PpsE acts in the cytoplasm, where PDIM is synthesized, the interaction suggests that MmpL7 domain 2 is accessible to the cytoplasm. We propose that MmpL7 acts as a scaffold to recruit PDIM synthetic machinery, forming a complex that coordinates lipid synthesis and transport.

## Results

### Identification of MmpL7–PpsE Interaction

**Figure 2:**
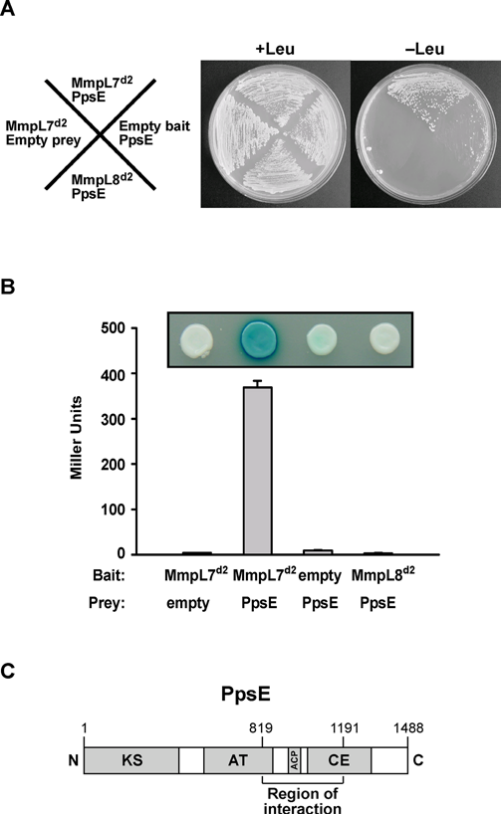
MmpL7 Domain 2 Interacts with the PDIM Synthesis Enzyme PpsE (A) Yeast two-hybrid reporter strains harboring the indicated bait and prey plasmids were streaked onto solid media with or without leucine. Growth on leucine-negative plates indicates a positive interaction. MmpL7^d2^, MmpL7 domain 2; MmpL8^d2^, MmpL8 domain 2. (B) The same strains as in (A) were transferred onto X-gal-containing indicator plates (inset), and reporter activity was quantified from liquid cultures using β-galactosidase assays. (C) Linear representation of full-length PpsE protein (1,488 amino acids) with the MmpL7 interaction region denoted. Catalytic domains of PpsE are also shown. ACP, acyl carrier protein; AT, acyl transferase; CE, condensing enzyme; KS, ketoacyl synthase.

To determine the mechanism of PDIM transport to the surface of *M. tuberculosis* bacilli, we first sought to identify components that interact with the two large non-TM domains of MmpL7 using a yeast two-hybrid approach (Figure 1B) [26]. We created two “bait” constructs to express either an MmpL7 domain 1 or an MmpL7 domain 2 fusion with the LexA DNA-binding protein in yeast. Although MmpL7 domain 1 activated transcription of both the *LEU2* and *lacZ* yeast reporters on its own, MmpL7 domain 2 expression did not auto-activate the reporters (Figure 2A and 2B) and thus was suitable for screening an *M. tuberculosis* genomic “prey” library for interactors. We identified positive library clones by selecting for growth on medium lacking leucine followed by blue–white screening on plates containing X-gal. Surprisingly, our screen identified an interaction between MmpL7 domain 2 and a 373-amino-acid fragment of PpsE, the enzyme required for the final step of phthiocerol synthesis (Figure 2).

A number of observations suggest that the interaction between MmpL7 domain 2 and PpsE is meaningful. First, PpsE was one of the strongest interactors identified in the screen as judged by blue color on indicator plates as well as quantitative β-galactosidase assays (>100-fold increase over controls; Figure 2B). Second, the interaction between MmpL7 domain 2 and PpsE is specific because PpsE did not interact with either LexA alone or with domain 2 of the homologous transporter MmpL8 (Figure 2A and 2B). Western blot analysis using anti-LexA antibodies indicated that the MmpL8 domain 2 and MmpL7 domain 2 baits were expressed at equivalent levels (data not shown). Finally, as PpsE is required for the synthesis of PDIM and MmpL7 is required for the transport of PDIM [8], the interaction between PpsE and MmpL7 suggested the intriguing possibility that PDIM synthesis and transport are coupled.

**Figure 3:**
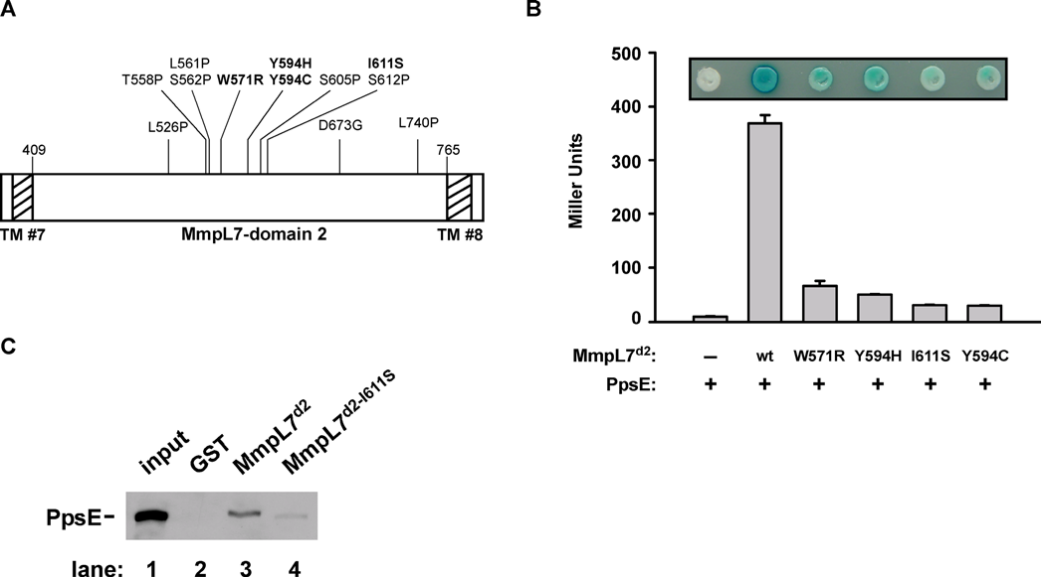
Identification of Residues in MmpL7 Domain 2 Required for PpsE Interaction (A) Twelve MmpL7 domain 2 mutants defective for PpsE binding were isolated in a reverse two-hybrid screen. These amino acid substitutions are displayed on a linear map of MmpL7 domain 2, with changes to amino acids other than proline or glycine in bold. Amino acid numbers correspond to positions in full-length MmpL7. TM domains 7 and 8 are denoted by hatched bars. (B) Yeast strains expressing the PpsE prey construct and various MmpL7 domain 2 bait plasmids were transferred onto X-gal indicator plates (inset), and reporter activity was quantified from liquid cultures by monitoring β-galactosidase activity. (C) Beads containing equal amounts of MmpL7 domain 2 and the I611S mutant were incubated with protein extracts containing myc-tagged PpsE and washed. Bound proteins were eluted and separated by SDS-PAGE, and PpsE was visualized by Western blot using anti-myc antibodies. GST-coated beads served as a negative control, and 1% of the protein extract added to the pulldown was loaded as a positive control (“input”).

To confirm the interaction between MmpL7 domain 2 and PpsE in vitro and test the interaction's role in vivo, we first sought to generate a mutant MmpL7 domain 2 that does not interact with PpsE. We used a reverse yeast two-hybrid approach [27] to identify single amino acid changes in MmpL7 domain 2 that disrupt its interaction with PpsE. We engineered our original MmpL7 domain 2 bait vector to include a C-terminal GFP fusion to easily avoid mutations that resulted in protein truncations. We randomly mutagenized MmpL7 domain 2 using error-prone PCR, introduced these constructs into yeast cells bearing the PpsE prey plasmid, and screened for colonies that were both white on X-gal indicator plates and GFP positive. Colonies with the desired phenotype were isolated and the bait plasmids were recovered, retested, and sequenced. Interestingly, of the 12 plasmids that contained mutations that led to single amino acid changes in MmpL7 domain 2, nine clustered in a 50-amino-acid region of the protein (Figure 3A). Eight of the mutations led to substitutions with proline or glycine, which are more likely to be structurally disruptive and possibly lead to global unfolding of the protein. The four remaining mutants, W571R, Y594H, Y594C, and I611S (amino acid numbers correspond to positions in full-length MmpL7) were reconstituted into the original bait vector lacking GFP and assayed for interaction both on X-gal plates and by liquid β-galactosidase assays (Figure 3B). Western blot analysis confirmed that these changes did not lead to differences in protein levels (data not shown). Since the I611S mutation is the most conservative mutation and leads to a large reduction in the interaction with PpsE, this mutant was chosen for subsequent analysis.

### MmpL7 Domain 2 Interacts with PpsE In Vitro

To independently test the interaction between MmpL7 domain 2 and PpsE identified in the two-hybrid screen, we performed in vitro GST pulldown experiments (Figure 3C). Glutathione agarose beads coated with MmpL7 domain 2 GST fusion protein were incubated with lysates from *M. smegmatis* cells expressing full-length, myc-tagged PpsE. PpsE interacted with beads coated with MmpL7 domain 2 but not with beads containing only GST protein (Figure 3C, lanes 2 and 3). Furthermore, the I611S mutation led to drastically reduced binding with PpsE (lane 4). Coomassie staining of proteins eluted from the beads demonstrated that equal amounts of MmpL7 domain 2 and the I611S mutant protein were present during the pulldown (data not shown). Importantly, while the I611S mutation had a large effect on PpsE binding, we consistently observed low-level residual binding both by GST pulldown and yeast two-hybrid assays. Taken together, our results demonstrate that MmpL7 domain 2 can specifically interact with PpsE in vitro.

### MmpL7 Domain 2 Inhibits PDIM Synthesis In Vivo

We next sought to determine whether the interaction between MmpL7 and PpsE occurs in vivo. Since MmpL7 is an integral membrane protein and PpsE is most likely present in the cytoplasm, we reasoned that MmpL7 domain 2 must be accessible to the cytoplasm in order to interact with PpsE. Overexpression of MmpL7 domain 2 in the cytoplasm would then act as a dominant negative by titrating PpsE away from endogenous full-length MmpL7.

**Figure 4:**
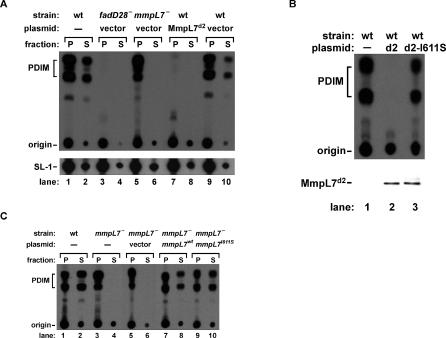
MmpL7 Domain 2 Acts as a Dominant Negative Inhibitor of PDIM Synthesis In Vivo (A) The indicated strains carrying either no plasmid (−), control vector (vector), or a plasmid with MmpL7 domain 2 under the control of the constitutive *groEL2* promoter (MmpL7^d2^) were labeled with ^14^C-propionate. Surface-exposed lipids (S) were extracted by resuspension in hexanes, and cell pellets (P) were harvested by centrifugation. Lipids from both fractions were extracted and separated by TLC under solvent conditions to separate PDIM (upper panel, keto and methoxy forms) and SL-1 (lower panel). (B) Top: lipids were extracted as in (A) from pellets of wild-type cells carrying either no plasmid (−), the MmpL7 domain 2 expression plasmid (d2), or the MmpL7 domain 2 expression plasmid with the I611S mutation (d2-I611S). Bottom: Western blot analysis was performed to confirm equivalent expression of wild-type MmpL7 domain 2 and the I611S mutant by using antibodies against the hemagglutinin epitope tag. (C) Complementation of an *mmpL7^−^ M. tuberculosis* strain with the wild-type (*mmpL7^wt^*) or the I611S mutant *mmpL7* (*mmpL7^I611S^*). Surface-exposed lipids (S) and lipids associated with the remaining cell pellet (P) were extracted and separated by TLC to resolve PDIM as in (A).

To this end we expressed MmpL7 domain 2 in the cytoplasm of wild-type cells, under the control of the constitutive *groEL2* promoter. To assay PDIM synthesis and transport, we labeled cells with ^14^C-propionate (which is incorporated into PDIM) and extracted surface-exposed lipids from the cell wall. In this procedure, radioactively labeled cells were resuspended in hexanes and then harvested by centrifugation to yield a supernatant fraction that contained surface-exposed lipids, and a pellet fraction that contained the remaining total lipids associated with the cell. Lipids from both fractions were extracted and separated by thin layer chromatography (TLC) in order to resolve PDIM (Figure 4A). As expected, in wild-type cells PDIM was present in both the pellet and supernatant fractions (Figure 4A, lanes 1 and 2), demonstrating that PDIM is both synthesized and transported. In a PDIM synthesis mutant, *fadD28* ^−^, PDIM was absent from both fractions (lanes 3 and 4), whereas in the PDIM transport mutant, *mmpL7* ^−^, PDIM was present only in the pellet fraction (lanes 5 and 6). Interestingly, overexpression of MmpL7 domain 2 in wild-type cells resulted in more than 98% inhibition of PDIM synthesis (lanes 7 and 8). Although overexpression of MmpL7 domain 2 did not cause any growth defects (data not shown), it was possible that it had a nonspecific effect on lipid synthesis. To confirm that overexpression of MmpL7 domain 2 specifically inhibits PDIM synthesis, we monitored SL-1, which like PDIM also incorporates propionic acid, in the same extracts and observed no differences in SL-1 synthesis or transport (Figure 4A).

The effect on PDIM synthesis was surprising, as *mmpL7* ^−^ cells are still able to synthesize PDIM; thus, a simple prediction would have been that a dominant negative form of MmpL7 would inhibit PDIM transport not synthesis. Although the mechanism of the dominant negative effect of domain 2 is unclear, the specific effect of MmpL7 domain 2 on PDIM synthesis, in addition to its specific interaction with PpsE, suggests that MmpL7 domain 2 interacts with PpsE in vivo.

To test whether the dominant negative effect of MmpL7 domain 2 on PDIM synthesis is indeed due to its interaction with PpsE, we overexpressed the I611S mutant form that fails to interact with PpsE. The I611S mutation was generated in the MmpL7 domain 2 overexpression construct and introduced into wild-type *M. tuberculosis* cells. Although MmpL7 domain 2 I611S was expressed at levels similar to those of the wild-type version, it was unable to inhibit PDIM synthesis to the same extent (Figure 4B). PDIM synthesis in this strain was approximately 50% of wild-type, consistent with the severely reduced, but not completely abolished, interaction of the I611S mutant MmpL7 domain 2 with PpsE. This result strongly suggests that MmpL7 domain 2 inhibits PDIM synthesis via direct inhibition of PpsE.

We reasoned that if MmpL7 interacts with PpsE in vivo to coordinately synthesize and transport PDIM, then an MmpL7 mutant that does not interact with PpsE may be defective for PDIM transport. To test this we introduced the I611S change in the context of full-length MmpL7 and expressed the wild-type and mutant forms of MmpL7 in an *mmpL7* ^−^ strain. We found that both forms were able to complement the PDIM transport defect (Figure 4C, lanes 8 and 10). Therefore, although the I611S mutation led to decreased interaction between MmpL7 domain 2 and PpsE in earlier experiments, in the context of full-length protein this mutation alone was not sufficient to decrease MmpL7 activity in vivo. Although the reason is unclear, this could be because MmpL7 has multiple interactions with PpsE, and perhaps with other members of the PDIM synthesis and transport machinery, that compensate for this mutation.

### Dominant Negative Effect of MmpL7 Domain 2 Requires MmpL7

**Figure 5:**
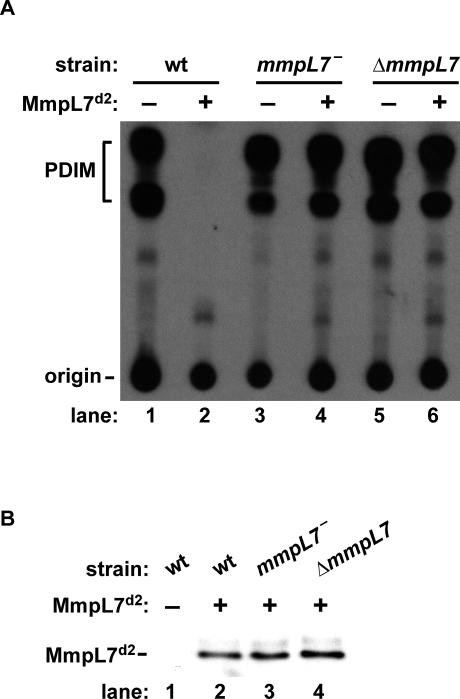
Dominant Negative Effect of MmpL7 Domain 2 Requires the Presence of Full-Length MmpL7 (A) Wild-type cells, an *mmpL7* transposon mutant (*mmpL7^ −^*), and a complete *mmpL7* knockout (Δ*mmpL7*) carrying either no plasmid (−) or the MmpL7 domain 2 expression construct (+). Labeled lipids were extracted from pellets as described in Figure 4 and separated by TLC to resolve PDIM. (B) Western blot of MmpL7 domain 2 showing that it is expressed at equivalent levels in the different *M. tuberculosis* strains.

A simple mechanism to account for the dominant negative effect of MmpL7 domain 2 expression is that the protein interacts directly with PpsE in the cytoplasm in such a way as to render the synthase inactive. Alternatively, because other RND family members act as trimers [22,23], it is possible that MmpL7 domain 2 may inhibit PpsE while in a complex with full-length MmpL7. To distinguish between these two possibilities, we tested whether the dominant negative effect of MmpL7 domain 2 requires the presence of full-length MmpL7. Interestingly, expression of MmpL7 domain 2 in *mmpL7^−^* transposon mutant cells failed to inhibit PDIM synthesis (Figure 5A, lane 4). Because the transposon mutant may express fragments of MmpL7, we also created an *M. tuberculosis* strain in which the full *mmpL7* gene was removed (Figure S1). Like the transposon mutant, the Δ*mmpL7* strain was unable to transport PDIM (Figure S1C) and was insensitive to the dominant negative effect of MmpL7 domain 2 expression (Figure 5A, lanes 5 and 6). Expression of MmpL7 domain 2 was similar in all three strains (Figure 5B, lanes 2–4). This finding demonstrates that the activity of cytoplasmically expressed MmpL7 domain 2 requires the presence of wild-type MmpL7. Taken together, our results suggest that MmpL7 domain 2 enters into a complex with endogenous MmpL7 that interacts with PpsE, trapping the synthase in an inactive state, thus inhibiting PDIM synthesis.

## Discussion

**Figure 6:**
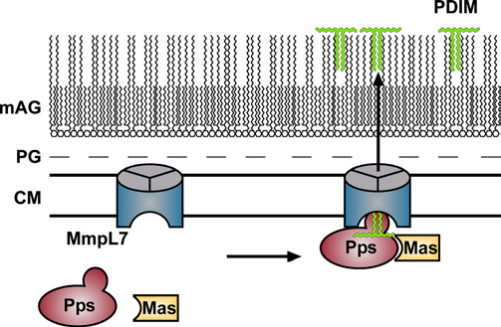
Model of PDIM Synthesis and Transport MmpL7 interacts with PpsE, a subunit of the Pps enzyme required for PDIM synthesis. We propose that MmpL7 acts as a scaffold to recruit PDIM synthesis machinery, including Pps and perhaps Mas, leading to coordinate synthesis and transport of PDIM across the cytoplasmic membrane (CM). Whether MmpL7, or other factors, are required for delivery of PDIM through the peptidoglycan (PG) and mycolyl-arabinogalactan (mAG) layers is unclear.

PDIM, like other polyketide lipids, is a key molecule in the pathogenesis of *M. tuberculosis*. In this study, we have identified a novel interaction between MmpL7, a protein required for PDIM transport, and PpsE, an enzyme required for PDIM synthesis. Overexpression of the interaction domain of MmpL7 causes a drastic defect in PDIM synthesis, suggesting that this domain interacts with PpsE in vivo and inhibits its activity. To our knowledge, this is the first report of an interaction between a synthetic enzyme and its cognate transporter. We propose that MmpL7 interacts with the PDIM synthetic machinery to form a complex that coordinately synthesizes and transports PDIM across the cell membrane (Figure 6).

Interestingly, the dominant negative effect of domain 2 on PDIM synthesis is dependent upon the presence of full-length MmpL7. This strongly suggests that domain 2 incorporates into a complex with endogenous MmpL7 and exerts its effect only in this context. Like AcrB, an RND transporter in *E. coli* [22,23], MmpL7 may normally act as a trimer or a higher order oligomer, and a hybrid complex of full-length MmpL7 with domain 2 may trap PpsE in an inactive state.

Since MmpL7 is dispensable for PDIM production it is curious that expression of domain 2 inhibits PDIM synthesis. We propose a simple model to reconcile this paradox. In a stepwise process of efficiently coordinating PDIM synthesis and export, MmpL7 may possess both inhibitory and activating activity on PDIM synthesis. For example, domain 2 may act to inhibit PpsE activity until the entire PDIM synthesis–transport complex is assembled, at which point the inhibition is relieved. Thus, MmpL7 domain 2 may exert its dominant negative effect by stabilizing the inhibitory state of this complex. In the absence of MmpL7, however, there is no inhibition or activation of PDIM synthesis, therefore PDIM synthesis is unaffected. This model would also explain why the I611S mutation, when reconstituted into full-length MmpL7, has no apparent effect. If the isoleucine residue is important for inhibition of PpsE, then the I611S mutation would not necessarily lead to a defect in PDIM synthesis or transport.

Since MmpL7 and AcrB share the defining features of RND transporters, it is tempting to draw parallels between the two proteins. Both contain 12 putative TM helices with non-TM loops between TM #1 and #2, and TM #7 and #8. In the crystal structure of AcrB, the non-TM domains are predicted to be periplasmic [22,23] and the TM domains form a central cavity that is accessible to the cytoplasm. TM prediction algorithms (TMPred, TMHMM) suggest that the non-TM domains of MmpL7 are also periplasmic, although no experimental data exist to validate this prediction. Since the interaction domain of MmpL7 lies between TM #7 and #8, in order to interact with PpsE, it must be accessible to the cytoplasm. There are a number of ways in which this could occur. First, like the glutamate transporter EAAT1 [28], the interaction domain of MmpL7 may be reentrant through the membrane and thus interact with PpsE. Alternatively, the PpsE protein may access the extracellular portion of MmpL7 via a central pore created by the MmpL7 TM domains. Finally, since MmpLs and AcrB are distantly related members of the RND permease family, the structure of MmpL7 may differ from AcrB, and the orientation of MmpL7 in the membrane may be such that domain 2 is located in the cytoplasm. Indeed, there are examples of evolutionarily related transporters with opposite membrane topology [29].

Since there is specificity in MmpL-mediated transport, it is attractive to speculate that this specificity may be in part due to the interaction with the cognate transporter. There is evidence in both *E. coli* and *Pseudomonas aeruginosa* that when the non-TM regions of two different RND permeases with different drug efflux specificities are swapped, the respective drug specificities are also switched [30,31]. We constructed analogous chimeras between MmpL7 and MmpL8; although these hybrids were expressed, they were nonfunctional (data not shown). Despite the negative result, this suggests that portions other than domains 1 and 2 are required for MmpL function.

Given the results presented here, we propose that MmpL proteins act not only as transporters but also as scaffolds to couple polyketide synthesis and secretion. This model may also provide a framework to explain the role of two other RND family transporters in polyketide synthesis. In *M. tuberculosis, mmpL8^−^* mutants are defective for SL-1 synthesis and accumulate a partially lipidated precursor SL_1278_ [9]. Originally, we proposed that MmpL8 may transport SL_1278_ across the cell membrane, where subsequent enzymatic steps would convert it to mature SL-1. However, in light of the interaction between MmpL7 and PpsE, it is now tempting to speculate that MmpL8 may similarly recruit a biosynthetic enzyme required to complete the synthesis of SL-1 prior to transport. Likewise, an RND transporter in *Streptomyces coelicolor,* ActII-ORF3, is also involved in the biogenesis of a polyketide, γ-actinorhodin [32]. Therefore, the coupling of polyketide synthesis and transport via interactions between synthases and cognate transporters may represent a general mechanism utilized by RND family members to efficiently export complex polyketides. This paradigm is reminiscent of protein secretion where newly synthesized polypeptides are co-translationally translocated across the membrane [33]. Coupling of synthesis and transport may be energetically favorable while promoting specificity and directionality in transport processes.

## Materials and Methods

### Strains and plasmids.

*M. tuberculosis* cells (Erdman strain) were cultured in 7H9 medium supplemented with 10% OADC, 0.5% glycerol, and 0.1% Tween-80, or on 7H10 solid agar medium with the same supplements except for Tween-80 [8]. Kanamycin (20 μg ml^−1^) and hygromycin (50 μg ml^−1^) were used where necessary. All strains and plasmids used in this study are described in Table S1.

### Construction of *M. tuberculosis* knockout strain.

The Δ*mmpL7* (MJM39) mutant strain was created by homologous recombination using specialized transducing phage phMJ1 [34]. This deletion replaced all 2,763 bp of *mmpL7* with a hygromycin resistance cassette, and correct replacement of the gene was confirmed by Southern blot analysis (Figure S1).

### Yeast two-hybrid assays.

The yeast two-hybrid screen was performed as described [26], and expression of bait proteins in yeast was confirmed by Western blotting using antibodies against LexA (kind gift of R. Brent). Bait constructs using MmpL7 domain 2 and MmpL8 domain 2 were created by PCR amplification and insertion into pEG202. The prey library was constructed using random Erdman genomic DNA fragments inserted into pjsc401. Positive interactors were selected on media lacking leucine and then screened by blue–white screening on agar plates containing X-gal (5-bromo-4-chloro-3-indolyl β-D-galactoside). β-galactosidase activity was assayed in yeast cells permeabilized with chloroform and sodium dodecyl sulfate as previously described [35]. For reverse two-hybrid assays, error-prone PCR was performed using Taq polymerase, 100 ng of the bait plasmid containing *mmpL7 domain 2* (pMJ2) as the PCR template, and 18 cycles of amplification. Mutagenesis rate was approximately one mutation per kilobase. Yeast homologous recombination was used to introduce the mutant MmpL7 domain 2 PCR product into the bait vector. GFP screening was performed visually by fluorescence microscopy.

### GST pulldown binding assays.

Recombinant MmpL7 domain 2–GST, MmpL7 domain 2–I611S–GST, and GST alone were expressed in *E. coli* strain DH5α by growing cultures to mid-logarithmic phase at 32 °C and inducing with 1 mM IPTG for 30 min. Cells were centrifuged and resuspended in 50 mM Tris, 1 mM EDTA, 150 mM NaCl, 1 mM PMSF, and 1 mM DTT, and lysed by sonication. Lysates were cleared by centrifugation at 12,000 *g* and incubated with Glutathione agarose beads (G 4510, Sigma, St. Louis, Missouri, United States) at 4 °C overnight. Beads were washed four times with PBS, 1 mM EDTA, and 0.5% Triton X-100, and then stored as a 50% slurry in 50 mM Tris, 1 mM EDTA, 500 mM NaCl, 20% glycerol, and 0.5% Triton X-100. PpsE-myc was expressed in *M. smegmatis,* and lysates were prepared by bead-beating cells into binding buffer (20 mM Tris, 1 mM EDTA, 150 mM NaCl, 1 mM PMSF, and 1 mM DTT). Lysates were incubated with 50 μl of protein-coated beads overnight at 4 °C, washed three times in binding buffer, and resuspended in SDS sample buffer. Samples were boiled to elute all proteins off the beads and resolved on 7.5% SDS-PAGE gels. PpsE-myc was detected by Western blotting using 9E10 monoclonal antibodies (kind gift of J. M. Bishop).

### Biochemical analysis of PDIM and SL-1.

*M. tuberculosis* cultures were labeled with ^14^C-propionate, which is incorporated into both PDIM and SL-1. Surface-exposed lipids were extracted by resuspending the cells in hexanes and gently sonicating [9]. Cell pellets, containing the remainder lipids, were harvested by centrifugation. Lipids from both fractions were extracted by the Bligh-Dyer method [36] and analyzed by separation on 10 cm × 10 cm HPTLC plates (Alltech Associates, Deerfield, Illinois, United States) by using either hexanes/ether (9:1) to resolve PDIM or chloroform/methanol/water (60:30:6) to resolve SL-1. Lipid spots on TLC plates were quantified using a phosphorimager.

### Protein preparation and analysis.

*M. tuberculosis* cells were grown into mid-logarithmic phase and harvested by centrifugation. Cell lysates were separated by SDS-PAGE using 12% polyacrylamide gels. Proteins were visualized by immunoblotting using monoclonal antibodies against the hemagglutinin epitope tag (HA.11, Covance, Berkeley, California, United States). Loading was normalized by total protein, and efficiency of transfer was confirmed by Ponceau S staining of the nitrocellulose membrane.

## Supporting Information

Figure S1 Creation of Δ*mmpL7* in *M. tuberculosis* by Specialized Transduction (A) Map of the *mmpL7* region in wild-type and the Δ*mmpL7* mutant showing the restriction sites and probe location for Southern blot. Genomic DNA from both wild-type and Δ*mmpL7* was digested with BamHI, and the blot was probed with a 1,029-bp 5′ flank to *mmpL7* revealing a 2,636-bp fragment for wild-type and a 4,700-bp fragment for the mutant.
(B) Southern blot of BamHI-digested genomic DNA from indicated strains.
(C) Surface-exposed lipids (S) and lipids associated with the remaining cell pellet (P) were labeled and extracted from wild-type and Δ*mmpL7* cells as described in Figure 4A and then separated by TLC to resolve PDIM.
(29 KB PDF)

Table S1 Strains and Plasmids Used in This Study (71 KB DOC)

## Abbreviations

Mas: mycocerosic acid synthase
PDIM: phthiocerol dimycocerosate
SL-1: sulfolipid-1
TLC: thin layer chromatography
TM: transmembrane
