# Supplementary figures and images for: Interaction between Polyketide Synthase and Transporter Suggests Coupled Synthesis and Export of Virulence Lipid in M. tuberculosis
Source: PLoS Pathog. 2005 Sep 30;1(1):e2. doi: 10.1371/journal.ppat.0010002 (PMC1238737; doi:10.1371/journal.ppat.0010002)

**A**

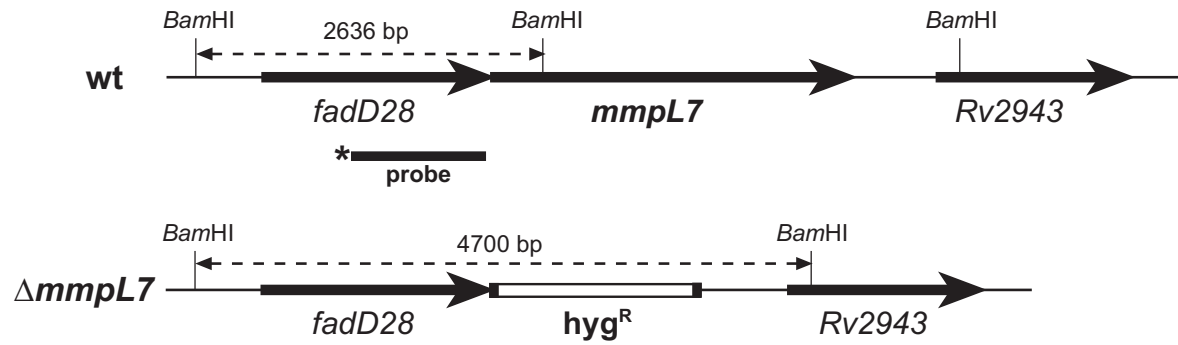

**B**

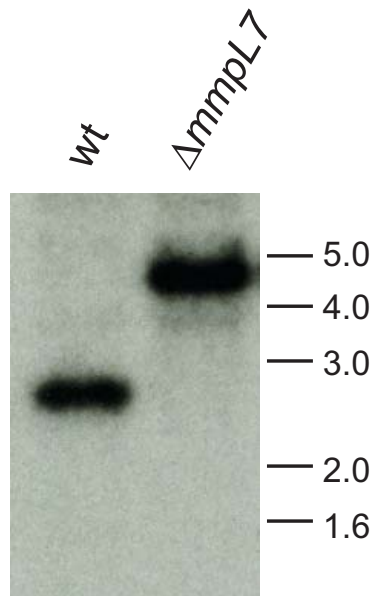

**C**

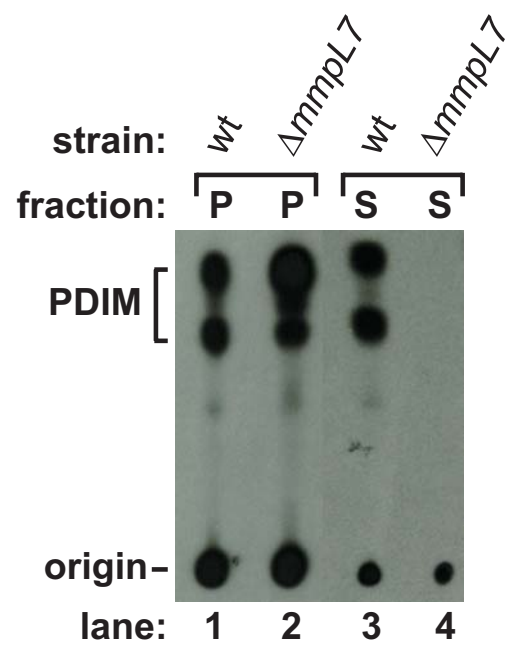

Figure S1

Supplement: Figure S1 — (A) Map of the mmpL7 region in wild-type and the ΔmmpL7 mutant showing the restriction sites and probe location for Southern blot. Genomic DNA from both wild-type and ΔmmpL7 was digested with BamHI, and the blot was probed with a 1,029-bp 5′ flank to mmpL7 revealing a 2,636-bp fragment for wild-type and a 4,700-bp fragment for the mutant. (B) Southern blot of BamHI-digested genomic DNA from indicated strains. (C) Surface-exposed lipids (S) and lipids associated with the remaining cell pellet (P) were labeled and extracted from wild-type and ΔmmpL7 cells as described in Figure 4A and then separated by TLC to resolve PDIM. (29 KB PDF) [file ppat.0010002.sg001.pdf]
