# Supplementary material for: Interaction between Polyketide Synthase and Transporter Suggests Coupled Synthesis and Export of Virulence Lipid in M. tuberculosis
Source: PLoS Pathog. 2005 Sep 30;1(1):e2. doi: 10.1371/journal.ppat.0010002 (PMC1238737; doi:10.1371/journal.ppat.0010002)
Supplement: Table S1 — (71 KB DOC) [file ppat.0010002.st001.doc]

TABLE S1: Strains and Plasmids used in this study

| **Strain or Plasmid** | **Genotype/Description** | **Source/ref.** |
| --- | --- | --- |
| *M. tuberculosis* |  |  |
| Erdman | wild-type | W. R. Jacobs, Jr. |
| mc^2^3106 | Erdman *fadD28::*Tn*5370*, Hyg^R^ | [1] |
| mc^2^3107 | Erdman *mmpL7::*Tn*5370*, Hyg^R^ | [1] |
| JCM96 | mc^2^3106 + pMV306.kan, Hyg^R^, Kan^R^ | This study |
| JCM98 | mc^2^3107 + pMV306.kan, Hyg^R^, Kan^R^ | This study |
| MJM20 | Erdman + pMJ35, Kan^R^ | This study |
| MJM21 | Erdman + pMJ35:2-100, Kan^R^ | This study |
| MJM2 | Erdman + pMV261.kan, Kan^R^ | This study |
| MJM3 | Erdman + pMJ10, Kan^R^ | This study |
| MJM4 | Erdman + pMJ13, Kan^R^ | This study |
| MJM22 | Erdman + pMJ10:2-100, Kan^R^ | This study |
| MJM36 | mc^2^3107 + pMJ35, Kan^R^ | This study |
| MJM39 | Erdman Δ*mmpL7*, Hyg^R^ | This study |
| MJM54 | MJM39 + pMJ35, Hyg^R^, Kan^R^ | This study |
| *Saccharomyces cerevisiae* |  |  |
| W303-1a | *MAT****a****; ura3-1; leu2-3,-112; his3-11,-15; trp1-1; ade2-1; can1-100* | P. Walter |
| EGY48 | *MAT****α****; trp1, his3, ura3, lexAop-LEU2* | R. Brent |
| *Plasmids* |  |  |
| pEG202 | LexA bait plasmid, *HIS3*, 2μm | [2] |
| pSH18-34 | 8 x *lexA*op*-lacZ* reporter, *URA3*, 2μm | [2] |
| pJG4-5 | *GAL1*prom-*GAL4*_AD_, *TRP1*, 2μm | [2] |
| pjsc401 | pJG4-5 + *Cla*I linker in MCS | [3] |
| pMJ2 | pEG202 + *mmpL7-domain 2* | This study |
| pMJ3 | pjsc401 + *ppsE* (a.a. 819-1191) | This study |
| pMJ6 | pEG202 + *mmpL8-domain 2* | This study |
| pMJ2:1-51 | pEG202 + *mmpL7-domain 2* W571R | This study |
| pMJ2:2-31 | pEG202 + *mmpL7-domain 2* Y594H | This study |
| pMJ2:2-100 | pEG202 + *mmpL7-domain 2* I611S | This study |
| pMJ2:3-1 | pEG202 + *mmpL7-domain 2* Y594C | This study |
| pMV306.kan | *int, oriE*, Kan^R^ | W. R. Jacobs, Jr. |
| pMV261.kan | *oriE*, *oriM*, groEL2 promoter, Kan^R^ | W. R. Jacobs, Jr. |
| pMJ13 | pMV261.kan + C-terminal in-frame *2xHA* tag, Kan^R^ | [4] |
| pMJ35 | pMJ13 + *mmpL7-domain 2* | This study |
| pMJ35:2-100 | pMJ13 + *mmpL7-domain 2* I611S | This study |
| pMJ10 | pMJ13 + *mmpL7* | This study |
| pMJ10:2-100 | pMJ13 + *mmpL7* I611S | This study |
| pGEX2T | P*tac*, lacI^Q^, N-terminal GST, Amp^R^ | Invitrogen (Carlsbad, CA) |
| pMJ4 | pGEX2T + *mmpL7-domain 2* | This study |
| pMJ74 | pGEX2T + *mmpL7-domain 2* I611S | This study |
| pMJ31 | pMV261.kan + C-terminal in-frame *myc* tag, Kan^R^ | [4] |
| pMJ32 | pMJ31 + *ppsE* | This study |

1. Cox JS, Chen B, McNeil M, Jacobs WR, Jr. (1999) Complex lipid determines tissue-specific replication of Mycobacterium tuberculosis in mice. Nature 402: 79-83.

2. Golemis E, Serebriiskii I, Finley RLJ, Kolonin MG, Gyuris J, et al. (1999) Interaction trap/two-hybrid system to identify interacting proteins. In: Ausubel FM, Brent R, Kingston RE, Moore DD, Seidman JG et al., editors. Current Protocols in Molecular Biology. New York: John Wiley & Sons.

3. Stanley SA, Raghavan S, Hwang WW, Cox JS (2003) Acute infection and macrophage subversion by Mycobacterium tuberculosis requires a novel specialized secretion system. Proc Natl Acad Sci USA 100: 13001-13006.

4. Converse SE, Cox JS (2005) A protein secretion pathway critical for Mycobacterium tuberculosis virulence is conserved and functional in Mycobacterium smegmatis. J Bacteriol 187: 1238-1245.
